# Differences in Breast and Colorectal Cancer Screening Adherence Among Women Residing in Urban and Rural Communities in the United States

**DOI:** 10.1001/jamanetworkopen.2021.28000

**Published:** 2021-10-04

**Authors:** Sanjay Shete, Yangyang Deng, Jackilen Shannon, Babalola Faseru, Deirdre Middleton, Ronaldo Iachan, Brittany Bernardo, Rajesh Balkrishnan, Sunny Jung Kim, Bin Huang, Morgan M. Millar, Bernard Fuemmler, Jakob D. Jensen, Jason A. Mendoza, Jinxiang Hu, DeAnn Lazovich, Linda Robertson, Wendy Demark-Wahnefried, Electra D. Paskett

**Affiliations:** 1 Division of Cancer Prevention, Department of Biostatistics, The University of Texas MD Anderson Cancer Center, Houston; 2 ICF, Rockville, Maryland; 3 Oregon Health & Sciences University, Portland State University School of Public Health, Portland; 4 Department of Population Health, University of Kansas Medical Center, Kansas City; 5 Division of Population Sciences, The Ohio State University Comprehensive Cancer Center, Columbus; 6 University of Virginia School of Medicine, Charlottesville; 7 Department of Health Behavior and Policy, Virginia Commonwealth University School of Medicine, Richmond; 8 Department of Cancer Biostatistics, University of Kentucky, Lexington; 9 University of Utah School of Medicine, Salt Lake City; 10 University of Utah College of Humanities, Salt Lake City; 11 Public Health Sciences Division, Fred Hutchinson Cancer Research Center, Seattle, Washington; 12 Department of Pediatrics, University of Washington, Seattle; 13 Department of Biostatistics and Data Science, University of Kansas Medical Center, Kansas City; 14 University of Minnesota School of Public Health, Minneapolis; 15 UPMC Hillman Cancer Center, Department of Medicine, University of Pittsburgh, Pittsburgh, Pennsylvania; 16 The University of Alabama at Birmingham, Birmingham; 17 Division of Cancer Prevention and Control, Department of Internal Medicine, The Ohio State University Comprehensive Cancer Center, College of Medicine, Columbus

## Abstract

**Importance:**

Screening for breast and colorectal cancer has resulted in reductions in mortality; however, questions remain regarding how these interventions are being diffused to all segments of the population. If an intervention is less amenable to diffusion, it could be associated with disparities in mortality rates, especially in rural vs urban areas.

**Objectives:**

To compare the prevalence of breast and colorectal cancer screening adherence and to identify factors associated with screening adherence among women residing in rural vs urban areas in the United States.

**Design, Setting, and Participants:**

This population-based cross-sectional study of women aged 50 to 75 years in 11 states was conducted from 2017 to 2020.

**Main Outcomes and Measures:**

Adherence to cancer screening based on the US Preventative Services Task Force guidelines. For breast cancer screening, women who had mammograms in the past 2 years were considered adherent. For colorectal cancer screening, women who had (1) a stool test in the past year, (2) a colonoscopy in the past 10 years, or (3) a sigmoidoscopy in the past 5 years were considered adherent. Rural status was coded using Rural Urban Continuum Codes, and other variables were assessed to identify factors associated with screening.

**Results:**

The overall sample of 2897 women included 1090 (38.4%) rural residents; 2393 (83.5%) non-Hispanic White women; 263 (9.2%) non-Hispanic Black women; 68 (2.4%) Hispanic women; 1629 women (56.2%) aged 50 to 64 years; and 712 women (24.8%) with a high school education or less. Women residing in urban areas were significantly more likely to be adherent to colorectal cancer screening compared with women residing in rural areas (1429 [82%] vs 848 [78%]; *P* = .01), whereas the groups were equally likely to be adherent to breast cancer screening (1347 [81%] vs 830 [81%]; *P* = .78). Multivariable mixed-effects logistic regression analyses confirmed that rural residence was associated with lower odds of being adherent to colorectal cancer screening (odds ratio [OR], 0.81; 95% CI, 0.66-0.99, *P* = .047). Non-Hispanic Black race was associated with adherence to breast cancer screening guidelines (OR, 2.85; 95% CI, 1.78-4.56; *P* < .001) but not colorectal cancer screening guidelines.

**Conclusions and Relevance:**

In this cross-sectional study, women residing in rural areas were less likely to be adherent to colorectal cancer screening guidelines but were similarly adherent to breast cancer screening. This suggests that colorectal cancer screening, a more recent intervention, may not be as available in rural areas as breast cancer screening, ie, colorectal screening has lower amenability.

## Key Points

**Question:**

Could the amenability index be extended to account for differences in breast and colorectal cancer screening adherence among women residing in urban vs rural communities in the United States?

**Findings:**

In a cross-sectional study of 2897 women from 11 US states, lower colorectal cancer screening adherence was found among rural-dwelling women compared with urban-dwelling women, but the prevalence of screening adherence for breast cancer was similar among women residing in urban and rural communities.

**Meaning:**

Rural colorectal cancer screening disparities could be explained by slower diffusion of colorectal cancer screening and present significant preventable public health challenges, which could be attenuated through effective interventions to increase diffusion of screening modalities.

## Teaser

This cross-sectional study compares the prevalence of breast and colorectal cancer screening adherence and identifies factors associated with screening adherence among women residing in rural vs urban areas.

## Introduction

While cancer rates have decreased consistently over the last several years, not all populations have realized similar declines.^1^ As more investigations have begun to focus on the rural United States, data are accumulating that suggest a much greater cancer burden in this population. While rural regions experience similar incidence of most cancers compared with urban areas, some cancers that can be prevented with regular screenings, such as cervical, colorectal, and lung cancer, have a higher prevalence in rural areas.^2^ Additionally, overall death rates due to cancer are higher in rural areas compared with urban areas.^2^ Rural is defined in many ways; however, by applying the Rural Urban Continuum Code (RUCC) definition,^3^ as accepted by the National Cancer Institute (NCI),^4^ 72% of the US land mass and 15% of the population, totaling 46.2 million US residents, are categorized as rural.^5^ Rural areas vary across the United States and include Appalachia, frontier lands, the Mississippi Delta, and prairie lands, among others.

Reasons for disparities in cancer rates in rural areas include a higher prevalence of poor health behaviors (eg, tobacco use, obesity, sedentary behavior)^6^ and poor access to cancer services (eg, screening, detection, and treatment).^7,8,9^ Tehranifar et al^10^ defined the amenability index, which reflects the degree that a cancer is amenable to medical intervention. They found that cancers most amenable to medical intervention demonstrated greater disparities in mortality by race and ethnicity,^10^ although they did not evaluate differences by geography. Potential reasons for these observed disparities in amenability are limited access to health care and treatment resources in some populations as well as slower diffusion of medical advances, when they are available, in certain populations, such as rural areas.^9,11^

The availability of modalities in rural areas allows for further examination of the amenability index in the context of rurality, as mammography is widely available and has been for many years^12^ while colorectal cancer screening, mainly colonoscopy, is less available in rural areas.^13^ Moreover, factors associated with adherence to breast and colorectal cancer screening guidelines can provide direction for local health care professionals and health departments to develop and implement interventions to increase the uptake of these screening tests to reduce disparities. The goal of this study, conducted in 11 states that span the nation, was to compare the prevalence of breast and colorectal cancer screening and identify factors associated with guideline adherence between rural-dwelling and urban-dwelling women aged 50 to 75 years. This analysis used data from the NCI Population Health Assessment in Cancer Center Catchment Area Initiative, which provided support for NCI-designated cancer centers to conduct research to better characterize populations within cancer center catchment areas.^14^ The goal of these initiatives was to facilitate cancer research collaborations and better understand health disparities, particularly at the local level, and included 2 rounds, conducted in 2017 to 2018 and 2019 to 2020.^15^

## Methods

### Data Source and Survey Methods

This pooled analysis included 11 population-based surveys from both round 1 and round 2 of the NCI Population Health Assessment. Round 1 sites were the University of Pittsburg Medical Center (UPMC) Hillman Cancer Center, University of Kentucky, and the Ohio State University. Round 2 sites were the University of Utah, Virginia Commonwealth University, University of Virginia, University of Minnesota, University of Alabama at Birmingham, Oregon Health & Science University, University of Kansas Cancer Center, and Fred Hutchinson Cancer Center. While most sites included both probability samples and nonprobability samples of key local subgroups, the pooled analysis only included the probability sample components of each site’s survey data set. While all data are from probability samples, the methods and modes varied. The surveys were completed by mail, telephone, in-person, or online. Table 1 describes the sample designs for each site (eg, mode: mail, telephone, in-person, web; sampling design: stratified random sample based on demographic characteristics or geographic distributions). The surveys shared common core measures that were developed collaboratively by the sites prior to fielding and used validated items from population-based surveys (Health Information National Trends Survey, Behavioral Risk Factor Surveillance System [BRFSS], National Health Interview Survey).^16^ At each site, protocols were reviewed, approved, and monitored by local institutional review boards. All participants provided written informed consent to participate in the study. This study was approved by the institutional review board at each of the participating sites: UPMC Hillman Cancer Center, University of Kentucky, The Ohio State University, University of Utah, Virginia Commonwealth University, University of Virginia, University of Minnesota, University of Alabama at Birmingham, Oregon Health & Science University, University of Kansas Cancer Center, and Fred Hutchinson Cancer Center. This study followed the Strengthening the Reporting of Observational Studies in Epidemiology (STROBE) reporting guideline.

**Table 1.** Summary Survey Design Features of the Surveys Implemented by the 11 Sites^a^

Cancer institute site	Participants, No.	Probability sample design	Catchment areas
**Round 1**			
Ohio State University Cancer Center	145^b^	Marketing Systems Group sample based on listed landline and consumer cellular phone numbers; stratified by age, sex, race, and geography	State of Ohio
University of Kentucky Markey Cancer Center	276	Random stratified ABS	54 Counties in eastern Kentucky designated as Appalachian
UPMC Hillman Cancer Center	247	Dual frame random digit dial sample	29 Counties in Western Pennsylvania
**Round 2**			
Fred Hutchison Cancer Research Center	221	Stratified random sample by sampling counties on urban, mostly urbanized, and mostly rural strata	13 Counties in western Washington state
University of Virginia Emily Couric Clinical Cancer Center	176	Geographically stratified ABS and random digit dialing (cellular)	81 Counties in Virginia, 13 counties in West Virginia
O’Neal Comprehensive Cancer Center at the University of Alabama at Birmingham	346	Geographically stratified random ABS	State of Alabama, age 50-80 years
The University of Kansas Cancer Center	532	Stratified random sample from 3 health systems and simple random sample from 1 health system file	State of Kansas and 18 counties in Missouri
Oregon Health & Science University Knight Cancer Institute	302	Rural-urban stratified random ABS	State of Oregon
University of Minnesota Masonic Cancer Center	208	Survey Sampling International mail survey	State of Minnesota
University of Utah	245	Stratified random sample of voter registration records linked to the Utah Population Database; recruitment modes, postal mail and telephone; data collection mode, paper	State of Utah
Virginia Commonwealth University Massey Cancer Center	199	Probability ABS with web or paper response options	63 Counties across Central and Eastern Virginia

Abbreviations: ABS, address-based sample; UPMC, University of Pittsburgh Medical Center.

^a^ The analysis was restricted to the probability sample components of the sites’ studies and to women aged 50 to 75 years.

^b^ Sample only included participants up to age 74 years.

### Study Population

Table 1 shows the sample size (for this analysis), sampling designs, survey methods, and catchment areas for each site. The aggregation of the data from the 11 sites required the harmonization of the variable names and response levels for all variables. For the purposes of analyzing screening adherence for breast cancer and colorectal cancer, the study population (2897 participants) was limited to women aged 50 to 75 years. Persons from rural areas were oversampled by most sites to improve the representation of the rural population.

### Dependent Variables

The 2 dichotomous dependent variables were focused on adherence to cancer screening based on the US Preventative Services Task Force guidelines.^17,18^ For breast cancer screening, women aged 50 to 74 years who had mammograms in the past 2 years were considered adherent (within guidelines). For colorectal cancer screening, adherent women aged 50 to 75 years were those who had (1) a stool test in the past year, (2) a colonoscopy in the past 10 years, or (3) a sigmoidoscopy in the past 5 years (only in round 1).

### Independent Variables

The study cancer centers’ surveys assessed a range of sociodemographic characteristics and behavioral variables, including urban or rural status, age, self-reported race (White, Black or African American, American Indian or Alaska Native, Asian Indian, Chinese, Filipino, Japanese, Korean, Vietnamese, other Asian, Native Hawaiian, Guamanian or Chamorro, Samoan, or other Pacific Islander) and ethnicity (Mexican, Mexican American, Chicano/a; Puerto Rican; Cuban; another Hispanic, Latino/a, or Spanish origin; or none of these), income, education, employment, financial security, primary source of health care coverage, marital status, body mass index (BMI; calculated as weight in kilograms divided by height in meters squared), cancer beliefs and perceptions, country of birth, source of health care, cost barrier to medical care, and smoking status. Not all sites collected information on country of birth, which accounts for the high percentage of missing values for this variable. To create the urban or rural status variable, we linked Federal Information Processing Standards codes with the 2013 RUCC developed by the United States Department of Agriculture.^3^ We dichotomized this 9-level variable as urban (RUCC 1-3) and rural (RUCC 4-9).

### Statistical Analysis

All analyses were conducted using unweighted data, as the combined data could not be considered representative of a particular population due to our pooled approach. Response categories were collapsed based on univariate tabulations for all potential factors associated with adherence (ie, independent variables) as well as for the 2 dependent variables. We used bivariate cross-tabulations and χ^2^ tests to examine whether there were significant associations between the independent variables and the dependent variables. Only those independent variables with significant associations with dependent variables (χ^2^ tests, *P* < .05) in bivariate analyses were included in the multivariable model. Missing data for these independent variables were addressed in different ways based on level of missing data. Three variables were excluded from the multivariable models because they had high percentage of missing data (>10% missing). These variables were country of birth and the 2 variables related to the usual place of health care. Variables with more than 8% but less than 10% missing data included missing as a category in the analyses. This included variables for medical cost, financial security, occupation, marital status, and income categories. Missing data values were then imputed for the other variables, which had less than 6% missing. We used multiple imputation to impute the missing values (PROC MI in SAS). More specifically, we used a fully conditional specification method^19,20^ that assumes the existence of a joint distribution for all variables. The number of imputations for PROC MI was 5.

We developed 2 multilevel mixed-effects logistic regression models with site as a random effect—1 for each of the cancer screening outcome measures—to identify factors associated with the most variance. The models included all independent variables selected from bivariate analysis. Multilevel mixed-effects logistic regression was used to model the association between rural residence and cancer screening adherence, adjusting for potential confounding due to sociodemographic characteristics and behavioral variables. Statistical significance was defined as a 2-sided *P* <.05. Statistical analysis was conducted with SAS software version 9.4 (SAS Institute).

## Results

The study population included 2897 women (1749 [61.6%] residing in urban communities; 1090 [38.4%] residing in rural communities). Table 2 shows the sociodemographic and behavioral characteristics of the study population stratified by urban and rural residence. Among the participants, 2393 (83.4%) were non-Hispanic White, 263 (9.2%) were non-Hispanic Black, and 68 (2.4%) were Hispanic.

**Table 2.** Descriptive Characteristics of 2897 Participants in All Cancer Center Catchment Areas

Characteristic	Participants, No. (%)			*P* value
	Urban	Rural	Total	
Age, y				
50-64	1023 (58.5)	577 (52.9)	1629 (56.2)	.004
65-75	726 (41.5)	513 (47.1)	1268 (43.8)	
Race and ethnicity				
Non-Hispanic White	1428 (82.6)	914 (84.6)	2393 (83.4)	.19
Non-Hispanic Black	169 (9.8)	92 (8.5)	263 (9.2)	
Hispanic	47 (2.7)	18 (1.7)	68 (2.4)	
Other^a^	85 (4.9)	57 (5.3)	143 (5.0)	
Income, $				
<20 000	234 (13.4)	223 (20.5)	472 (16.3)	<.001
20 000-49 999	328 (18.8)	320 (29.4)	663 (22.9)	
50 000-99 999	449 (25.7)	290 (26.6)	749 (25.9)	
≥100 000	334 (19.1)	123 (11.3)	461 (15.9)	
Missing	404 (23.1)	134 (12.3)	552 (19.0)	
Education				
≤High school	336 (19.4)	349 (32.1)	712 (24.8)	<.001
Post–high school trainings	484 (27.9)	373 (34.3)	873 (30.3)	
≥College	913 (52.7)	366 (33.6)	1291 (44.9)	
Employment status				
Employed	665 (38.0)	321 (29.4)	1001 (34.6)	<.001
Retired	602 (34.4)	444 (40.7)	1069 (36.9)	
Reported disability	132 (7.5)	153 (14.0)	297 (10.3)	
Other	151 (8.6)	106 (9.7)	264 (9.1)	
Missing	199 (11.4)	66 (6.1)	266 (9.2)	
Financial security on present income				
Living comfortably	651 (37.2)	372 (34.1)	1043 (36.0)	<.001
Getting by	413 (23.6)	373 (34.2)	802 (27.7)	
Finding it difficult	169 (9.7)	148 (13.6)	328 (11.3)	
Finding it very difficult	72 (4.1)	64 (5.9)	140 (4.8)	
Missing	444 (25.4)	133 (12.2)	584 (20.2)	
Primary source of health coverage				
Uninsured	72 (4.2)	38 (3.7)	112 (4.0)	<.001
Medicare	592 (34.7)	468 (45.7)	1081 (38.8)	
Medicaid	78 (4.6)	58 (5.7)	146 (5.2)	
Private or employee based	882 (51.6)	410 (40.0)	1309 (47.0)	
Other	84 (4.9)	50 (4.9)	136 (4.9)	
Marital status				
Married or living as married	843 (48.2)	651 (59.7)	1527 (52.7)	<.001
Not married	706 (40.4)	378 (34.7)	1108 (38.3)	
Missing	200 (11.4)	61 (5.6)	262 (9.0)	
BMI				
<25, Under or normal weight	447 (32.3)	275 (29.1)	735 (30.8)	.28
25 to <30, Overweight	408 (29.4)	293 (31.0)	720 (30.2)	
≥30, Obesity	531 (38.3)	376 (39.8)	930 (39.0)	
“It seems like everything causes cancer”				
Strongly or somewhat agree	905 (52.8)	667 (62.5)	1609 (56.7)	<.001
Strongly or somewhat disagree	810 (47.2)	401 (37.5)	1231 (43.3)	
“There’s not much you can do to lower your chances of getting cancer”				
Strongly or somewhat agree	293 (16.8)	262 (24.0)	570 (19.7)	<.001
Strongly or somewhat disagree	1305 (74.6)	772 (70.8)	2097 (72.4)	
Missing	151 (8.6)	56 (5.1)	230 (7.9)	
“There are so many different recommendations about preventing cancer, it’s hard to know which ones to follow”				
Strongly or somewhat agree	1176 (68.9)	812 (75.6)	2034 (71.7)	.001
Strongly or somewhat disagree	530 (31.1)	262 (24.4)	803 (28.3)	
“When I think about cancer, I automatically think about death”				
Strongly or somewhat agree	860 (50.1)	620 (57.9)	1517 (53.4)	<.001
Strongly somewhat disagree	855 (49.9)	451 (42.1)	1326 (46.6)	
“Cancer is most often caused by a person’s behavior or lifestyle”				
Strongly or somewhat agree	683 (44.6)	486 (48.6)	1193 (46.1)	.05
Strongly or somewhat disagree	847 (55.4)	514 (51.4)	1393 (53.9)	
Smoking status				
Current	172 (10.0)	139 (13.1)	317 (11.2)	.008
Former	473 (27.5)	250 (23.5)	738 (25.9)	
Never	1077 (62.5)	676 (63.5)	1789 (62.9)	
In the past 12 mo, was there a time when you needed to see a doctor, but could not because of cost?				
Yes	152 (10.2)	131 (12.9)	287 (11.2)	.03
No	1343 (89.8)	886 (87.1)	2280 (88.8)	
Is there a place that you usually go to when you are sick or need advice about your health?				
Yes	1056 (78.6)	620 (80.5)	1686 (79.3)	.42
There is no place	49 (3.6)	21 (2.7)	71 (3.3)	
There is more than 1 place	238 (17.7)	129 (16.8)	370 (17.4)	
What kind of place do you go most often?				
Clinic or health center	241 (16.8)	272 (27.7)	534 (21.6)	<.001
Doctor’s office or HMO	1085 (75.7)	642 (65.3)	1760 (71.1)	
Hospital emergency department	17 (1.2)	7 (0.7)	24 (1.0)	
Hospital outpatient department	9 (0.6)	6 (0.6)	15 (0.6)	
Some other place	47 (3.3)	25 (2.5)	74 (3.0)	
Do not go to 1 place most often	35 (2.4)	31 (3.2)	66 (2.7)	
Born in the United States				
Yes	892 (95.5)	405 (97.1)	1308 (96.0)	.16
No	42 (4.5)	12 (2.9)	54 (4.0)	

Abbreviations: BMI, body mass index (calculated as weight in kilograms divided by height in meters squared); HMO, health maintenance organization.

^a^ Other race includes American Indian or Alaska Native, Asian Indian, Chinese, Filipino, Japanese, Korean, Vietnamese, other Asian, Native Hawaiian, Guamanian or Chamorro, Samoan, and other Pacific Islander.

Women residing in rural areas compared with those residing in urban areas reported significantly lower levels of income (<$20 000: 223 [20.5%] vs 234 [13.4%]; $20 000-$49 999: 320 [29.4%] vs 328 [18.8%]); education (≤high school: 349 [32.1%] vs 336 [19.4%]); employment prevalence (321 [29.4%] vs 665 [38.0%]); and financial security (found it difficult to make ends meet on present income: 148 [13.6%] vs 169 [9.7%]). Also, rural-dwelling women less frequently used a doctor’s office or HMO compared with urban-dwelling women (642 [65.3%] vs 1085 [75.7%]). On the other hand, women residing in rural areas, compared with women residing in urban areas, were more likely to be receiving Medicare (468 [45.7%] vs 592 [34.7%]); be married (651 [59.7%] vs 843 [48.2%]); currently smoke (139 [13.1%] vs 172 [10.0%]); and needed to have visited a doctor in the past 12 months but could not because of cost (131 [12.9%] vs 152 [10.2%]).

Rural-dwelling women were more likely to hold the following beliefs compared with urban-dwelling women: “It seems like everything causes cancer” (667 [62.5%] vs 905 [52.8%]), “There’s not much you can do to lower your chances of getting cancer” (262 [24.0%] vs 293 [16.8%]), “There are so many different recommendations about preventing cancer, it’s hard to know which ones to follow” (812 [75.6%] vs 1176 [68.9%]), “When I think about cancer, I automatically think about death” (620 [57.9%] vs 860 [50.1%]), and “Cancer is most often caused by a person’s behavior or lifestyle” (486 [48.6%] vs 683 [44.6%]). There were no significant differences among rural-dwelling vs urban-dwelling women in BMI (obesity: 376 [39.8%] vs 531 [38.3%]) and country of birth (born in US: 405 of 417 [97.1%] vs 892 of 934 [95.5%]).

Table 3 shows the unadjusted cancer screening prevalence and association with rural or urban status. Women in both urban (1347 [81%; 95% CI, 79%-83%]) and rural (830 [81%; 95% CI, 78%-83%]) areas were equally likely to be breast cancer screening adherent, whereas women residing in urban areas were significantly more likely to be adherent to colorectal cancer screening (1429 [82%; 95% CI, 80%-84%]) compared with women residing in rural areas (848 [78%; 95% CI, 75%-80%]).

**Table 3.** Unadjusted Association of RUCCs With Breast and Colorectal Cancer Screening Adherence

RUCC	Breast cancer screening			Colorectal cancer screening		
	No. (% [95% CI])		*P* value	No. (% [95% CI])		*P* value
	Yes	No		Yes	No	
1-3, Urban	1347 (81 [79-83])	317 (19 [17-21])	.78	1429 (82 [80-84])	320 (18 [16-20])	.01
4-9, Rural	830 (81 [78-83])	201 (19 [17-22])		848 (78 [75-80])	242 (22 [20-25])	

Abbreviation: RUCC, Rural Urban Continuum Code.

Multivariable mixed-effects logistic regression analyses identified higher breast cancer screening adherence among non-Hispanic Black women vs non-Hispanic White women (odds ratio [OR], 2.85; 95% CI, 1.78-4.56; *P* < .001); those with primary source of health coverage through Medicare (OR, 2.84; 95% CI, 1.81-4.47; *P* < .001), Medicaid (OR, 2.58; 95% CI, 1.47-4.52; *P* = .001), a private or employee-based plan (OR, 3.80; 95% CI, 2.45-5.88; *P* < .001), and other insurance (OR, 2.07; 95% CI, 1.17-3.65; *P* = .01) vs women without insurance; among retired vs employed women (OR, 1.40; 95% CI, 1.06-1.86; *P* = .02); and among women who answered no to the question “In the past 12 months was there a time when you needed to see a doctor, but could not because of cost?” vs those who answered yes (OR, 1.56; 95% CI, 1.13-2.14; *P* = .006). Factors associated with lower breast cancer screening adherence included those who were getting by on present income vs women living comfortably on present income (OR, 0.69; 95% CI, 0.52-0.91; *P* = .008); and currently smoking vs never smoked (OR, 0.56; 95% CI, 0.41-0.75; *P* < .001) (Table 4).

**Table 4.** Multivariable Mixed-Effects Logistic Regression Model Identifying Factors Associated With Breast Cancer Screening Adherence

Variable	Odds ratio (95% CI)	*P* value
Race and ethnicity		
Non-Hispanic Black	2.85 (1.78-4.56)	<.001
Hispanic	1.31 (0.69-2.50)	.41
Other^a^	0.76 (0.50-1.16)	.20
Non-Hispanic White	1 [Reference]	NA
Income, $		
20 000-49 999	1.00 (0.71-1.41)	.99
50 000-99 999	1.11 (0.75-1.65)	.60
≥100 000	1.17 (0.72-1.91)	.52
<20 000	1 [Reference]	NA
Primary source of health coverage		
Medicare	2.84 (1.81-4.47)	<.001
Medicaid	2.58 (1.47-4.52)	.001
Private or employee based	3.80 (2.45-5.88)	<.001
Other	2.07 (1.17-3.65)	.01
Uninsured	1 [Reference]	NA
Education		
Post–high school trainings	1.15 (0.88-1.51)	.30
≥College	1.19 (0.90-1.58)	.21
≤High school	1 [Reference]	NA
Employment status		
Retired	1.40 (1.06-1.86)	.02
Reported disability	0.99 (0.67-1.45)	.95
Other	1.11 (0.77-1.60)	.57
Employed	1 [Reference]	NA
Financial security on present income		
Getting by	0.69 (0.52-0.91)	.008
Finding it difficult	0.77 (0.53-1.12)	.17
Finding it very difficult	0.94 (0.55-1.59)	.82
Living comfortably	1 [Reference]	NA
Marital status		
Not married	0.89 (0.71-1.13)	.34
Married or living as married	1 [Reference]	NA
“When I think about cancer, I automatically think about death”		
Strongly or somewhat disagree	0.97 (0.79-1.19)	.78
Strongly or somewhat agree	1 [Reference]	NA
Smoking status		
Current	0.56 (0.41-0.75)	<.001
Former	0.91 (0.72-1.15)	.42
Never	1 [Reference]	NA
In the past 12 mo was there a time when you needed to see a doctor, but could not because of cost?		
No	1.56 (1.13-2.14)	.006
Yes	1 [Reference]	NA

Abbreviation: NA, not applicable.

^a^ Other race includes American Indian or Alaska Native, Asian Indian, Chinese, Filipino, Japanese, Korean, Vietnamese, other Asian, Native Hawaiian, Guamanian or Chamorro, Samoan, and other Pacific Islander.

For colorectal cancer screening, multivariable mixed-effects logistic regression analyses found lower adherence among rural vs urban residents (OR, 0.81; 95% CI, 0.66-0.99; *P* = .047). Factors associated with higher colorectal cancer screening adherence included those aged 65 and older vs those aged 50 to 64 years (OR, 1.57; 95% CI, 1.16-2.12; *P* = .004); those with income between $50 000 and $99 999 and those with income $100 000 and greater vs those with income less than $20 000 ($50 000-$99 999: OR, 1.81; 95% CI, 1.24-2.66, *P* = .002; ≥$100 000: OR, 1.68; 95% CI, 1.06-2.66; *P* = .03); those with primary source of health coverage through Medicare (OR, 2.34; 95% CI, 1.43-3.83; *P* < .001), Medicaid (OR, 2.00; 95% CI, 1.15-3.49; *P* = .01), and private or employee-based plans (OR, 1.99; 95% CI, 1.30-3.06; *P* = .002) vs women without insurance; retired women and women with a disability vs employed women (retired: OR, 1.48; 95% CI, 1.12-1.96; *P* = .01; with disability: OR, 2.20; 95% CI, 1.46-3.31; *P* < .001); and those who answered no to the question “In the past 12 months was there a time when you needed to see a doctor, but could not because of cost?” vs those who answered yes (OR, 1.42; 95% CI, 1.04-1.92; *P* = .03) (Table 5).

**Table 5.** Multivariable Mixed-Effects Logistic Regression Model Identifying Factors Associated With Colorectal Cancer Screening Adherence

Variable	Odds ratio (95% CI)	*P* value
RUCC code		
4-9, Rural	0.81 (0.66-0.99)	.047
1-3, Urban	1 [Reference]	NA
Age, y		
≥65	1.57 (1.16-2.12)	.004
50-64	1 [Reference]	NA
Race and ethnicity		
Non-Hispanic Black	1.27 (0.86-1.87)	.23
Hispanic	1.24 (0.64-2.39)	.52
Other^a^	0.75 (0.50-1.13)	.17
Non-Hispanic White	1 [Reference]	NA
Income, $		
20 000-49 999	1.22 (0.88-1.70)	.24
50 000-99 999	1.81 (1.24-2.66)	.002
≥100 000	1.68 (1.06-2.66)	.03
<20 000	1 [Reference]	NA
Primary source of health coverage		
Medicare	2.34 (1.43-3.83)	<.001
Medicaid	2.00 (1.15-3.49)	.01
Private or employee based	1.99 (1.30-3.06)	.002
Other	1.56 (0.89-2.76)	.12
Uninsured	1 [Reference]	NA
Education		
Post–high school trainings	1.17 (0.90-1.52)	.24
≥College graduate	1.30 (0.99-1.71)	.06
≤High school	1 [Reference]	NA
Employment status		
Retired	1.48 (1.12-1.96)	.01
Reported disability	2.20 (1.46-3.31)	<.001
Other	1.15 (0.81-1.61)	.44
Employed	1 [Reference]	NA
Financial security on present income		
Getting by	0.87 (0.67-1.14)	.31
Finding it difficult	1.00 (0.70-1.45)	.98
Finding it very difficult	0.95 (0.58-1.57)	.84
Living comfortably	1 [Reference]	NA
Marital status		
Not married	0.94 (0.74-1.18)	.58
Married or living as married	1 [Reference]	NA
“It seems like everything causes cancer”		
Strongly or somewhat disagree	1.08 (0.88-1.33)	.46
Strongly or somewhat agree	1 [Reference]	NA
“There’s not much you can do to lower your chances of getting cancer”		
Strongly or somewhat disagree	1.04 (0.81-1.34)	.76
Strongly or somewhat agree	1 [Reference]	NA
“When I think about cancer, I automatically think about death”		
Strongly or somewhat disagree	1.14 (0.93-1.39)	.22
Strongly or somewhat agree	1 [Reference]	NA
Smoking status		
Current	0.77 (0.57-1.03)	.07
Former	1.26 (0.99-1.60)	.06
Never	1 [Reference]	NA
In the past 12 mo was there a time when you needed to see a doctor, but could not because of cost?		
No	1.42 (1.04-1.92)	.03
Yes	1 [Reference]	NA

Abbreviations: NA, not applicable; RUCC, Rural Urban Continuum Code.

^a^ Other race includes American Indian or Alaska Native, Asian Indian, Chinese, Filipino, Japanese, Korean, Vietnamese, other Asian, Native Hawaiian, Guamanian or Chamorro, Samoan, and other Pacific Islander.

## Discussion

Disparities in cancer incidence and mortality are evident in rural areas. Several studies have documented decreased cancer screening rates in rural communities.^21,22,23,24^ One reason for these disparities centers on the slower diffusion of medical advances, like cancer screening, in rural areas. The goal of this study was to assess the prevalence of breast and colorectal cancer screening among age-eligible women in rural vs urban populations in 11 states and the factors associated with being adherent to screening guidelines. As hypothesized, rural residence made a difference in not being up to date with colorectal cancer screening but not breast cancer screening, even in multivariable analyses. This suggests that the diffusion of colorectal cancer screening modalities might be slower in rural areas.

This finding is important, as previous studies looking at diffusion of interventions as a cause of disparities have only identified this trend by race, ethnicity, and age,^10,25^ not by rural status. Our large, widespread geographic study is, to our knowledge, the first to identify this association. Even though this was a cross-sectional study, this difference is important and suggests that public health interventions should focus efforts to increase colorectal cancer screening in rural areas. It is important to note that while colonoscopy is reported as favored and the most recommended modality by primary care practitioners,^26,27^ it may not be the best solution in rural communities, where travel and access barriers are pronounced for colorectal cancer screening.^28,29^ Newer stool-based tests, like fecal immunohistochemical tests, might be more acceptable to rural residents and can address issues with respect to access barriers in rural communities.^30^ These tests should be considered and implemented widely in rural areas, as they can be mailed to patients and returned by mail, reducing even the need for a face-to-face visit, especially relevant now in the COVID-19 era. All insurers cover these tests, thus making them affordable. However, health care professionals would have to establish call and recall mechanisms for those who do not adhere as well as those who have positive test results to insure prompt and proper follow-up.

Other findings are worth discussing. Given that an inability to leave work is cited by 1 of 4 persons as a barrier to colonoscopy and sigmoidoscopy,^31^ it is easy to understand why individuals of retirement age may be more adherent to colorectal cancer screening guidelines. Similar associations between age and screening have been reported for mammography^32,33,34^; however, no significant associations were observed in the current study. The possible reasons could be the distinct sampling designs used; continued adherence to screening over time, as studied in the Beaber et al^32^; and the fact that younger women were included in the studies by Narayan et al^34^ (ages 40 to 74 years) and Guo et al^33^ (all ages 18 years and older), in which the prevalence of nonadherence was highest among women younger than 50 years.

Participants who had health insurance were 2 to 3 times more likely to be adherent to breast cancer and colorectal cancer screening compared with those without insurance. Increase in health care coverage as a result of the Patient Protection and Affordable Care Act, which included elimination of copay for preventive health services including breast and colorectal cancer screening, has been well documented and found to be associated with an increase in screening rates for breast and colorectal cancer.^35,36,37^ In Canada, where socialized medicine is practiced, positive and significant associations have been observed between colorectal cancer screening and advancing age, which corroborate the findings in our study.^38^ These findings suggest that access to health care coverage may be an incentive for adherence to breast and colorectal cancer screening.

Another interesting finding in our study was that family income was not significantly associated with breast cancer screening adherence but was associated with colorectal cancer screening adherence for family income more than $50 000 compared with family income less than $20 000. It is possible that the association of health care coverage with breast cancer screening adherence is independent of the influence of family income. This is an area for further investigation.

Knowledge and beliefs about cancer may have an influence on an individual’s preventative behaviors.^15^ There is some evidence that rural individuals tend to endorse more fatalistic beliefs about cancer, such as everything causes cancer, there is not much you can do to lower chances of developing cancer, and it is difficult to know what screening recommendations to follow.^39,40^ These findings are similar to those in the current study, where cancer fatalism has been implicated in an individual’s decision-making about preventative cancer screenings given that their perception is that cancer is out of an individual’s control.

In the current analyses, after accounting for rural and urban residence, fatalistic beliefs did not contribute to cancer screening behaviors, suggesting that the role of geography may be more strongly associated with screening behaviors than fatalistic beliefs. The findings of the current study somewhat differed from those in the study by Moss et al,^40^ as there were no differences in breast cancer screening rates between rural-dwelling and urban-dwelling women. However, rural women (78%) were less likely to be up to date with colorectal cancer screening than their urban counterparts (82%); a 4% difference is not only statistically significant but also meaningful within the context of public health, given that the magnitude of difference exceeds that of the goals set for Healthy People 2030 vs those set for 2020, ie, 3.9%. There are few studies that have examined rurality as a determinant of fatalistic beliefs about cancer.^39^ There is a need to better understand the geographic differences in cancer related beliefs and their relationship to preventative behaviors.

Additionally, while we might expect differences in breast cancer screening by race, given that non-Hispanic Black individuals have much worse cancer outcomes than non-Hispanic White individuals, as suggested by 2018 data from the National Center for Health Statistics (NCHS) that shows a mortality rate ratio of 1.41 between non-Hispanic Black and non-Hispanic White individuals,^41,42^ data from the current study indicate that adherence to mammography screening was significantly higher in non-Hispanic Black women than among non-Hispanic White women, and this difference was not explained by rurality or other covariates. While counterintuitive, our results regarding mammography screening parallel those reported by the NCHS in 2018.^42^ While some studies suggest that higher screening rates reported by non-Hispanic Black individuals may be an artifact associated with overreporting,^43,44^ other studies suggest that the Patient Protection and Affordable Care Act has been instrumental in countering low screening rates that were reported historically among minority racial and ethnic groups.^45^ Should this be the case, we would anticipate changes in disparities in mortality in coming years. Our findings support prior reports of colorectal cancer screening being adversely associated with income and insurance status and skewed toward an older population.^46,47,48^ However, we did not see significant differences in colorectal cancer screening rates in non-Hispanic Black women vs non-Hispanic White women, as has been previously reported.^49^ Others have reported patient fear, patient and physician knowledge, or barriers to screening and access to health care services as reasons for disparities in non-Hispanic Black vs non-Hispanic White women for colorectal cancer screening.^50^ It is possible that accounting for factors such as insurance status, rurality (one marker of access), and cancer beliefs attenuated the association with race in our study.

While the rural health disparities observed in this study present substantial public health challenges, it is possible to improve cancer outcomes through appropriate public health interventions. For example, a 2020 study^51^ found that a mailed motivational message with contact information to request a free at-home fecal immunochemical screening test (compared with a mailed reminder to schedule a screening appointment) effectively improved adherence to screening guidelines in a rural community. An evaluation of patient navigation program in rural Georgia found the program dramatically improved the odds of adherence with colorectal screening guidelines among participants.^52^ Building on successes like these will help to address the screening needs of rural women described in this paper.

### Strengths and Limitations

This study benefitted from the ability to pool data from 11 surveys due to the coordination in development of the survey instruments prior to fielding and technical support to pool the data for analyses. It would not have been possible to conduct an analysis of this small subgroup limited by age and sex in any one site alone due to the limitations of sample size. That being said, there still are limitations based on the relative homogeneity of the sample with regard to some characteristics, such as race and ethnicity, given that the pooled sample was still largely composed of non-Hispanic White women. Thus, while larger subgroups, such as non-Hispanic Black women, could be compared with non-Hispanic White women, comparisons of other subgroups (eg, Hispanic or Asian women) was not feasible. The inclusion of multiple data sources also introduced some constraints. Because the samples were drawn from disparate populations, it was not practical to create survey weights for representation of a distinct population, and caution should be exercised when making inferences from these results to broader populations. Furthermore, data on colorectal cancer screening modality were not available for each site; therefore, we could not compare urban-rural difference by screening options. However, the study data still provide valid inference for the association of urban and rural geographies with screening.

Another limitation may be that while we dichotomized the rural and urban categories, there may be differences even within these smaller groups. Those living in an area with a RUCC score of 4 may be quite different from those with a RUCC score of 9; however, for our purposes RUCCs 4 to 9 were combined and labeled as rural. More granular analyses in the future may demonstrate differences within these dichotomized categories. Another limitation may be that the research was conducted by cancer research centers, and therefore, those with greater trust in these institutions may have been more willing to respond to the surveys. Also, we relied on self-reports of screening, which we acknowledge may be biased; however, other large surveys, eg, the BRFSS,^53^ use self- reports of screening. Our estimates of 78% for colorectal cancer screening and 82% for breast cancer screening were not far from BRFSS estimates of 70% and 78%, respectively.^53^ Moreover, studies have indicated that self-report overestimates utilization compared with medical record data for colorectal cancer screening more than breast screening.^44^ Regardless, the findings need to be evaluated with these limitations in mind.

## Conclusions

In this study, rural-dwelling women had lower rates of adherence to colorectal cancer screening compared with women living in urban areas. However, both groups of women had similar rates of adherence to breast cancer screening. These findings suggest that colorectal cancer screening may not be as available in rural areas as breast cancer screening.

## Notes

Supplement.
Nonauthor Collaborators
