# Supplementary material for: Differences in Breast and Colorectal Cancer Screening Adherence Among Women Residing in Urban and Rural Communities in the United States
Source: JAMA Netw Open. 2021 Oct 4;4(10):e2128000. doi: 10.1001/jamanetworkopen.2021.28000 (PMC8491105; doi:10.1001/jamanetworkopen.2021.28000)
Supplement: Supplement. — Nonauthor Collaborators [file jamanetwopen-e2128000-s001.pdf]

Supplemental Online Content: Nonauthor Collaborators

*Indicates required information. Only first name, last name, and suffix will appear in PubMed.

***Group Name(s): The Rural Workgroup of the Population Health Assessment in Cancer Center Catchment Areas Initiative**							
***First Name and Middle Initial(s)**	***Last Name**	***Suffix (eg, Jr, III)**	Academic Degrees	Institution	Location (city, state/province, country)	Role or Contribution, eg, chair, principal investigator	Group (if more than 1 Group listed in the byline) and/or Subgroup (eg, Steering Committee)
Abigail	Foulds		PhD	UPMC Hillman Cancer Center	Pittsburgh, Pennsylvania, USA		
Andrew	Mrkva		MA	UPMC Hillman Cancer Center	Pittsburgh, Pennsylvania, USA		
Todd	Bear		PhD	UPMC Hillman Cancer Center	Pittsburgh, Pennsylvania, USA		
Jian-Min	Yuan		MD, PhD	UPMC Hillman Cancer Center	Pittsburgh, Pennsylvania, USA		
Amy	Ferketich		PhD	Ohio State University	Columbus, Ohio, USA		
Jay	Fisher		PhD	Ohio State University	Columbus, Ohio, USA		
Timothy	Huerta		PhD	Ohio State University	Columbus, Ohio, USA		
Ann	McAlearney		ScD	Ohio State University	Columbus, Ohio, USA		
Darrell	Gray		MD	Ohio State University	Columbus, Ohio, USA		
Chasity	Washington		MPH	Ohio State University	Columbus, Ohio, USA		
Darla	Fickle		MA	Ohio State University	Columbus, Ohio, USA		
Heather	Aker		MPH	Ohio State University	Columbus, Ohio, USA		
Stephanie	Dickinson		MS	Indiana University	Blooomington, Indiana, USA		
Chen	Lyu		PhD	Indiana University	Blooomington, Indiana, USA		
Sina	Kianersi		DVM	Indiana University	Blooomington, Indiana, USA		
Heather	Anderson		BA	University of Utah	Salt Lake City, Utah, USA		
Ken	Smith		PhD	University of Utah	Salt Lake City, Utah, USA		
Debra	Ma		MBA	University of Utah	Salt Lake City, Utah, USA		
Helen	Lillie		PhD	University of Utah	Salt Lake City, Utah, USA		
Sean	Upshaw		PhD	University of Utah	Salt Lake City, Utah, USA		
Bassam	Dahman		PhD	Virginia Commonwealth University	Richmond, Virginia, USA		
David	Wheeler		PhD	Virginia Commonwealth University	Richmond, Virginia, USA		
Tamas	Gal		PhD	Virginia Commonwealth University	Richmond, Virginia, USA		
Albert	Ksinan		PhD	Virginia Commonwealth University	Richmond, Virginia, USA		
Bonny	Morris		PhD	Virginia Commonwealth University	Richmond, Virginia, USA		
Carrie	Miller		PhD	Virginia Commonwealth University	Richmond, Virginia, USA		
Elizabeth	Do		PhD	Virginia Commonwealth University	Richmond, Virginia, USA		
Kendall	Fugate-Laus		MS	Virginia Commonwealth University	Richmond, Virginia, USA		
Westley	Fallavollita		BS	Virginia Commonwealth University	Richmond, Virginia, USA		
Gordon	Ginder		MD	Virginia Commonwealth University	Richmond, Virginia, USA		

Supplemental Online Content: Nonauthor Collaborators

*Indicates required information. Only first name, last name, and suffix will appear in PubMed.

***First Name and Middle Initial(s)**	***Last Name**	***Suffix (eg, Jr, III)**	Academic Degrees	Institution	Location (city, state/province, country)	Role or Contribution, eg, chair, principal investigator	Group (if more than 1 Group listed in the byline) and/or Subgroup (eg, Steering Committee)
Robert	Winn		MD	Virginia Commonwealth University	Richmond, Virginia, USA		
Thomas	Loughran		MD	University of Virginia	Charlottesville, Virginia, USA		
Mona	Fouad		MD	University of Alabama Birmingham	Birmingham, Alabama, USA		
Sejong	Bae		PhD	University of Alabama Birmingham	Birmingham, Alabama, USA		
Isabel	Scarinci		PhD	University of Alabama Birmingham	Birmingham, Alabama, USA		
Monica	Baskin		PhD	University of Alabama Birmingham	Birmingham, Alabama, USA		
Casey	Daniel		PhD	University of Alabama Birmingham	Birmingham, Alabama, USA		
Claudia	Hardy		MPA	University of Alabama Birmingham	Birmingham, Alabama, USA		
Paige	Farris		MSW	Oregon Health & Science University	Portland, Oregon, USA		
Motomi	Mori		PhD	Oregon Health & Science University	Portland, Oregon, USA		
Zhenzhen	Zhang		PhD	Oregon Health & Science University	Portland, Oregon, USA		
Stacy	McCrea-Robertson		MS	University of Kansas Cancer Center	Kansas City, Kansas, USA		
Allen	Greiner		MD	University of Kansas Cancer Center	Kansas City, Kansas, USA		
David	Doody		MS	Fred Hutch/University of Washingtor	Seattle, Washinton, USA		
Roy	Jensen		MD, PhD	University of Kansas Cancer Center	Kansas City, Kansas, USA		
Edward	Ellerbeck		MD, MPH	University of Kansas Cancer Center	Kansas City, Kansas, USA		
Ronald	Chen		MD, MPH	University of Kansas Cancer Center	Kansas City, Kansas, USA		
Hope	Krebill		RN, BSN,	University of Kansas Cancer Center	Kansas City, Kansas, USA		
Danny	Kurz		MPH	University of Kansas Cancer Center	Kansas City, Kansas, USA		
Noelle	Voges		MA	University of Virginia	Charlottesville, Virginia, USA		
George	Batten		PhD	University of Virginia	Charlottesville, Virginia, USA		
Roger	Anderson		PhD	University of Virginia	Charlottesville, Virginia, USA		
Lindsay	Hauser		MA	University of Virginia	Charlottesville, Virginia, USA		
Thomas	Guterbock		PhD	University of Virginia	Charlottesville, Virginia, USA		
Rej	Desai		MS	University of Virginia	Charlottesville, Virginia, USA		
Benjamin	Haaland		PhD	University of Utah	Salt Lake City, Utah, USA		
